# Unusual radiographic appearance of fecalith leading to acute abdomen in a patient consuming iron: a case report

**DOI:** 10.1097/MS9.0000000000003053

**Published:** 2025-02-28

**Authors:** Niharika Pathak, Megha Thapa, Sushil Sah, Prakash Pandey, Padam Raj Joshi

**Affiliations:** a Bajhang District Hospital, Bajhang, Nepal; b Institute of Medicine, Tribhuvan University, Kathmandu, Nepal; c Nepal Army Institute of Health Sciences, Kathmandu, Nepal; d Rukum District Hospital, Rukum, Nepal; e Sailaja Acharya Cardiac Centre, Biratnagar, Nepal

**Keywords:** acute abdomen, case report, fecalith, iron intake, radiography

## Abstract

**Background and importance:**

Fecalith is common clinical condition, delay in diagnosis and management can lead to life threatening complications.

**Case presentation:**

We are reporting the case of an 18-year- old lady which have unusual radiographic appearance of fecalith which was diagnosed in rural emergency setting and managed with simple conservative approach.

**Clinical discussion:**

Fecalith can also be present in background of iron consumption without chronic constipation and anatomical abnormality. Large fecalith can have confusing radiographic picture.

**Conclusion:**

Fecalith can present with acute abdomen and confusing radiographic picture. It can lead to life-threatening bowel complications but can be managed conservatively with early diagnosis.

## Introduction and background

Fecal impaction is a frequent cause of emergency department visits^[1]^. Although, it is an easily manageable condition, it can lead to serious complications such as bowel perforation and may become life-threatening^[2,3]^. Most of the time it is related to seed bezoars consumption in elderly and children in background of chronic constipation but iron consumption leading large fecalith formation is rarely reported.^[4]^ Here, we report a case of acute abdomen under SCARE 2023 criteria (Supplementary Digital Content, file 1, http://links.lww.com/MS9/A742) caused by a fecalith which was successfully managed.

**Highlights**

- Fecalith can present in emergency with severe abdominal pain, that is refractory to multiple analgesia.
- Fecalith can have unusual radiographic picture of well- defined, homogenous, round and large radio-opacity.
- Possible associated with history of iron intake without other gastrointestinal disorder and fiber intake.

## Methods

We report the case based on surgical case report guideline (SCARE) 2023^[5]^.

## Case presentation

We report a case of an 18-year-old unmarried female who presented to our center with sudden onset of colicky abdominal pain. The patient described the pain similar to bearing down effort explaining like “something coming out of the vagina” which progressively worsened over 24 hours and was associated with mild abdominal distension. She had not passed stool for the past 4 days and had not passed flatus for the last 24 hours. Prior to this, she gave a history of consumption of iron tablets (250 mg) once daily for 7 days self-prescribed with oral contraceptive pills following which she started having constipation. There is no history of similar episodes in the past, no history of any neurological disorder, chronic constipation, or any other medical condition.

On examination, the vital signs were stable but she reported pain score of 10/10 in visual analogue scale, and the abdomen was soft with mild hypogastric tenderness. Initially, bowel or ureteric colic was suspected and we started treatment with hyoscine and intravenous ketorolac. However, the pain persisted necessitating the addition of intravenous tramadol, which also failed to provide significant relief.

**Figure 1.**
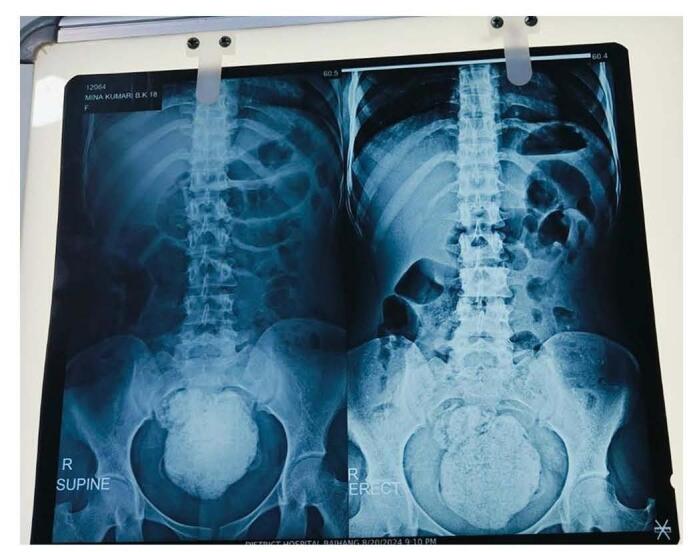
X-ray abdomen showing erect and supine views showing well defined 6.6*6 cm round opacity is present in the pelvis.

**Figure 2.**
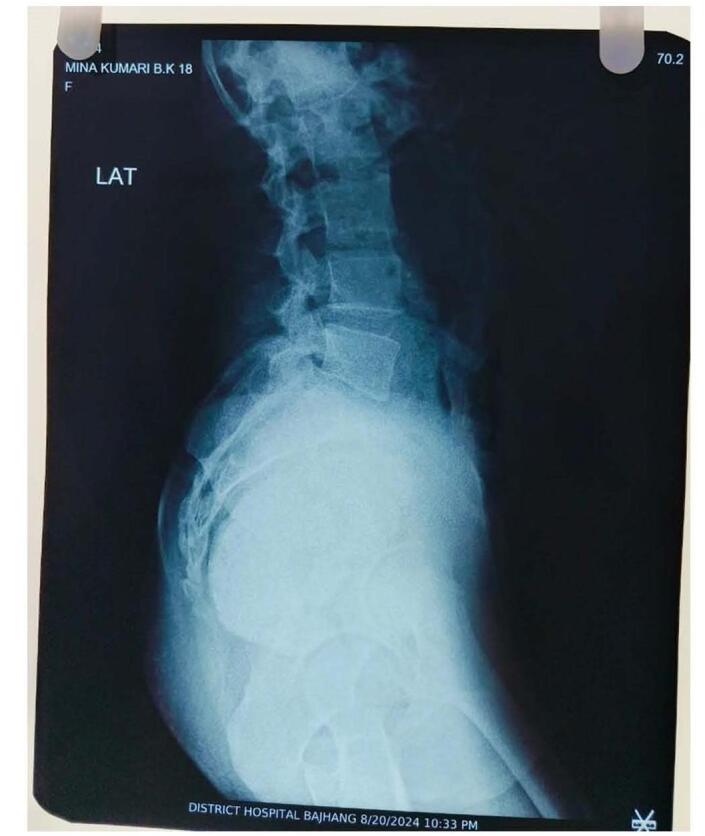
X-ray lateral view showing the 6 × 5 cm radiopacity lying in front of the sacral bone.

**Figure 3.**
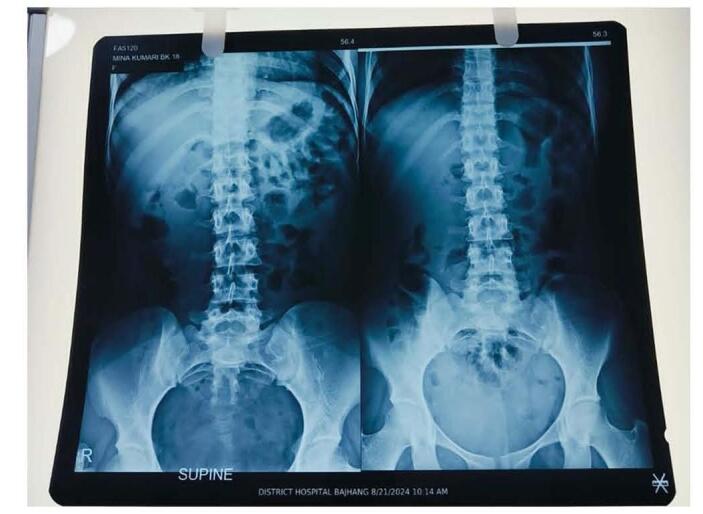
Post treatment X-ray abdomen erect and supine views showing no abnormality.

Her hemoglobin, iron profile and electrolytes were within normal limit. Abdominal radiograph revealed a large, well-defined, homogenous, spherical radiopacity in front of the sacral bone and behind the pubic symphysis along with mild dilation of proximal bowel wall as shown in Figs 1 and 2. This was followed by an ultrasonography which showed that the uterus and urinary bladder were normal and there was a well-defined hyperechoic lesion behind the uterus with posterior acoustic shadowing. On per rectal examination, the perineum appeared normal with an intact anal opening, but a well-defined mass was felt, which stained the gloves with a substance resembling black mud. By making the working diagnosis of fecalith, we managed the case by digital fragmentation of the lesion followed by 200 mL of soap water enema after which she passed the stool and the pain subsided. Repeat abdominal X-ray and ultrasonography produced normal findings (Fig. 3). Later the patient was discharged with oral medication and she was doing well with no any further complain even at 1 week and 3 months of follow up.

## Discussion

Fecalith may present in an emergency department with acute abdomen, typically in a patient with a history of chronic constipation. However, its presentation with severe abdominal pain along with bearing down sensation in a young female without a known co-morbidity is very unusual. There is no such case reported in literature.^[1]^ Previously it has been reported that high fiber diet like seeds is commonly associated with fecal impaction. In this case, in contrary to fiber or seed intake there is history of intake of iron tablets. Although, the association of iron consumption and fecalith formation is not well established, its association needs to be established by well-designed study. There are studies, which mentioned its association with constipation, which can be potential mechanism for fecalith formation.^[6-8]^

Our patient presented with severe abdominal pain with bearing down effort which was not controlled by first line analgesia, potentially leading us to an incorrect diagnosis. Abdominal radiography is one of the most commonly used diagnostic modality in children and adolescents and it helps in diagnosis of fecalith. We found one reported case of large fecalith which was associated with an anorectal anomaly but no any case of a large fecalith in absence of chronic anorectal condition^[9]^. Although, radiography is helpful in diagnosis of fecaliths, the radiographic findings in our case were unusual, i.e. a large well defined homogenous radiopacity in pelvic cavity. It resembles pelvic calcified mass or large bladder stone. In past, fecal impaction with tiny fecalith was reported as comparatively smaller and heterogenous multiple radio density. This created uncertainty in confirming diagnosis in our case. Though, CT scan would have helped to diagnose and detect complications. In setting, where a CT scan is unavailable, a high clinical suspicion combined with radiographic and ultrasonic studies can aid in diagnosis^[10]^

Digital fragmentation combined with soap water enema helps in successful management and prevention of fatal complications^[11]^. Fecalith if not managed in time can potentially be life threatening^[12]^.

## Conclusion

Without background of chronic constipation in young individual large fecalith can present with acute abdomen and confusing radiographic picture. It can lead to life-threatening bowel complications but can be managed conservatively with early diagnosis.

### Limitations

Three months of follow up may be shorter for concluding exact outcome of case.

## Supplementary Material

## Data Availability

Not applicable.

## References

[1] Hussain ZH Whitehead DA Lacy BE. Fecal impaction. Curr Gastroenterol Rep 2014;16:1-7. 10.1007/s11894-014-0404-2 25119877

[2] Mou D Atkinson R Valero M. Rare but life-threatening complication of fecalith. BMJ Case Rep 2018;2018:bcr–2018–226174. 10.1136/bcr-2018-226174 PMC6047690 29991555

[3] Serrano Falcón B Barceló López M Mateos Muñoz B. Fecal impaction: a systematic review of its medical complications. BMC Geriatr 2016;16:1-8. https://pubmed.ncbi.nlm.nih.gov/26754969/. 26754969 10.1186/s12877-015-0162-5 PMC4709889

[4] Sahebzamani FM Berarducci A Murr MM. Malabsorption anemia and iron supplement induced constipation in post-roux-en-Y gastric bypass (RYGB) patients. J Am Assoc Nurse Pract 2013;25:634–40. 24170670 10.1002/2327-6924.12079

[5] Sohrabi C Mathew G Maria N. The SCARE 2023 guideline: updating consensus Surgical CAse REport (SCARE) guidelines. Int J Surg 2023;109:1136–40. 37013953 10.1097/JS9.0000000000000373 PMC10389401

[6] Eitan A Bickel A Katz IM. Fecal impaction in adults: report of 30 cases of seed bezoars in the rectum. Dis Colon Rectum 2006;49:1768–71. 17036204 10.1007/s10350-006-0713-0

[7] Muller C Muller S Sissoko A. Radio-opaque fecal impaction and pseudo-occlusion in a dialyzed patient taking lanthanum carbonate. Hemodial Int 2012;16:556–58. 22118504 10.1111/j.1542-4758.2011.00647.x

[8] Kamath S Parveen RS Hegde S. Daily versus alternate day oral iron therapy in iron deficiency anemia: a systematic review. Naunyn Schmiedebergs Arch Pharmacol 2024;397:2701–14. 37979057 10.1007/s00210-023-02817-7

[9] Jalil O Jones H Stephenson BM. Faecaloma in ano. Ann R Coll Surg Engl 2012;94:e68–e69. 22391354 10.1308/003588412X13171221501069 PMC5827240

[10] Reuchlin-Vroklage LM Bierma-Zeinstra S Benninga MA. Diagnostic value of abdominal radiography in constipated children: a systematic review. Arch Pediatr Adolesc Med 2005;159:671–78. 15997002 10.1001/archpedi.159.7.671

[11] Chakravartty S Chang A Nunoo-Mensah J. A systematic review of stercoral perforation. Colorectal Dis 2013;15:930–35. 23331762 10.1111/codi.12123

[12] Corban C Sommers T Sengupta N. Fecal impaction in the emergency department: an analysis of frequency and associated charges in 2011. J Clin Gastroenterol 2016;50:572–77. 26669560 10.1097/MCG.0000000000000458

